# Stabilization of Amylopectin–Xyloglucan Water-in-Water Pickering Emulsions by Yeast Cells

**DOI:** 10.1021/acsomega.6c05131

**Published:** 2026-08-29

**Authors:** Cassiano Pires, Lilian Fernanda Martins do Amaral, Natalia Brasil Posselt Costa, Joslaine Jacumazo, Jaqueline Carneiro, Lazhar Benyahia, Rilton Alves de Freitas

**Affiliations:** † BIOPOL, Center for Development and Innovation in Materials and Biomaterials-CDIM, Curitiba 80210-170, Brazil; ‡ IMMM, UMR 6283, CNRS—Le Mans Université, Avenue Olivier Messiaen, Le Mans 72085, France

## Abstract

Pickering water-in-water (W/W) emulsions stabilized by biocompatible particles represent a promising strategy for the design of functional soft materials. In this study, we investigate the ability of *Saccharomyces cerevisiae*, in both viable (Y-AC) and thermally inactivated (Y-IN) forms, to stabilize amylopectin–xyloglucan (AMP–XG) W/W emulsions, providing the first report of yeast-based Pickering stabilization in this polymer–polymer system. Physicochemical characterization, including FTIR, ζ-potential measurements, and potentiometric titration, revealed that thermal inactivation induces a reorganization of the cell wall, increasing the fraction of titratable sites (from 23% to 50%) while preserving the overall acid–base balance and net negative surface charge. The ζ-potential was highly sensitive to ionic strength: the addition of 1 mmol L^–1^ NaCl reduced its magnitude by approximately 50%, whereas 20 mmol L^–1^ nearly suppressed electrostatic repulsion. Stability assays showed that Y-IN at 6.0 × 10^4^ cells mL^–1^ provided effective interfacial coverage and delayed phase separation. However, elevated ionic strength (20 mmol L^–1^ NaCl), induced yeast migration toward the AMP-rich phase, compromising emulsion stability. Confocal microscopy confirmed that viable cells also strongly adsorb at the polymer–polymer interface but undergo similar salt-induced migration at high ionic strength. Contact angle measurements further indicated a shift in wettability toward the dispersed phase, increasing from 89° ± 11° to 116° ± 10° (Y-IN) and from 114 ± 18° to 130° ± 7° (Y-AC) with increasing salt concentration. Overall, these findings demonstrate that yeast particles can function as natural stabilizers for AMP–XG W/W emulsions, with ionic strength acting as a key parameter in modulating interfacial adsorption and phase affinity.

## Graphical abstract

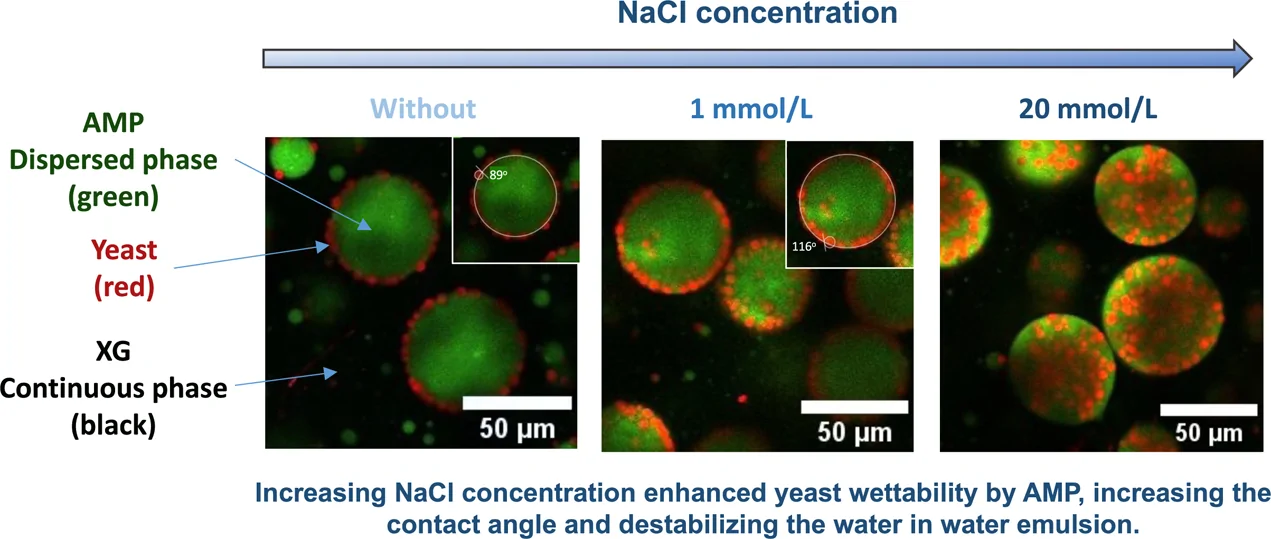

## 1 Introduction

Water-in-water (W/W) emulsions are colloidal dispersions composed of two immiscible aqueous polymer phases that arise from thermodynamic phase separation due to the incompatibility of at least two water-soluble polymers in solution.^1^ Unlike conventional oil–water systems, W/W emulsions cannot be efficiently stabilized by molecular surfactants, as their short amphiphilic chains are ineffective at anchoring to the extended, low-tension interfaces characteristic of these systems.^2^ A promising alternative is the use of colloidal particles to form Pickering emulsions (PE), where the irreversible interfacial adsorption of solid particles creates a mechanical barrier against droplet coalescence and phase separation.^1,3^ PEs are stabilized by solid particles that irreversibly adsorb at the interface between immiscible liquids, generating a mechanical barrier that enhances long-term stability.^4^ Due to their reduced susceptibility to aggregation and Ostwald ripening, PEs have garnered increasing interest in the food, pharmaceutical, and cosmetic industries, particularly driven by the demand for natural stabilizers.^5,6^

A wide range of particles can stabilize PE, including inorganic and polymeric materials that differ in size, morphology, surface charge, and pH sensitivity. However, some inorganic particles may raise biocompatibility and environmental concerns, motivating the search for sustainable, food-grade alternatives.^7^ In this context, biopolymers have proven versatile, with stabilization efficiency strongly dependent on molecular structure and interfacial interactions. For instance, β-lactoglobulin microgels stabilize amylopectin–xyloglucan (AMP–XG) emulsions at pH 5 by localizing at the interface, with fibrillar and spherical morphologies exhibiting distinct efficiencies.^8,9^ Similarly, cellulose allomorph nanoparticles display contrasting behaviors: rod-like cellulose nanocrystals (type I) interact with XG to promote gelation, whereas spherical cellulose nanospheres (type II) preferentially migrate to the AMP–XG interface, effectively stabilizing the system.^10^

The use of Pickering W/W emulsions has been explored in microbial systems. Recent studies have reported the simultaneous inoculation of *Lactobacillus helveticus* and *Saccharomyces cerevisiae* using W/W emulsions stabilized by microcrystalline cellulose, highlighting the feasibility of these systems for controlled coculture and microbial structuring.^11,12^ In this context, microorganisms are not merely passive cargo but can actively contribute to emulsion stabilization. Indeed, microorganisms can act as effective stabilizing agents in PEs. Their efficiency depends on size, shape, and surface chemistry, which influences adsorption, packing, and interfacial coverage.^13^ Geometric features (e.g., rods, cocci, ellipsoids) and surface properties such as roughness, charge, and composition, affect stability by modulating adhesion and desorption dynamics. Gram-positive bacteria, for example, possess surface proteins and a thick peptidoglycan layer that reduce interfacial tension, while extracellular polysaccharides promote additional adsorption at the interface.^13,14^

Beyond bacteria, *S. cerevisiae* and other yeasts have attracted growing attention due to their recognized safety (GRAS), industrial applicability, and probiotic potential. Early studies reported that dried *S. cerevisiae* cells decrease oil–water interfacial tension via hydrophilic membrane constituents.^15^ More recent approaches demonstrated that hydrophobically modified yeast cells can stabilize oil-in-water PEs,^16^ while yeast cell wall fragments and protein-based nanoparticles contribute to the formation of stable high-internal-phase emulsions.^17^ Comparative analyses further indicated that *S. cerevisiae* may surpass lactic acid bacteria in maintaining emulsion stability^14^ and that even nonviable microorganisms can organize distinctive W/W microstructures with tunable rheology.^18^ Beyond stabilization, the use of viable microbial cells in PE introduces additional functionality, since probiotic microorganisms could act as biostabilizers while conferring nutritional or health benefits, creating dual-function systems where the stabilizer itself offers biological benefits.^13^

Previous research demonstrated a whole-cell-based Pickering interfacial biocatalysis system, where living microorganisms functioned simultaneously as emulsifiers and biocatalysts.^19^ Inspired by this approach, the present study explores viable *S. cerevisiae*, a food-grade yeast, as a natural stabilizer in nongelling, noncharged AMP–XG W/W emulsions. Although there are studies with W/W systems involving *S. cerevisiae*, in these cases, the yeast did not act directly as a Pickering particle.^11,12^ We hypothesize that the unique surface architecture of *S. cerevisiae* enables interfacial adsorption and stabilization of polymer–polymer interfaces without the need for conventional emulsifiers. Moreover, the presence of metabolically active yeast may confer additional probiotic functionality, leading to bioactive emulsions with potential applications in food and pharmaceutical systems. The influence of ionic strength (NaCl) on yeast interfacial behavior and emulsion stability is also addressed, providing insights into how cell–biopolymer interactions can be tuned to design novel bioactive soft interfaces. To date, the literature lacks studies specifically investigating AMP–XG W/W systems stabilized by *S. cerevisiae* as Pickering particles, underscoring the novelty of this research.

## 2 Materials and Methods

### 2.1 Materials

Amylopectin (AMP; 3.0 × 10^7^ g mol^–1^) was purchased from Sigma-Aldrich (USA). Xyloglucan (XG; 1.2 × 10^6^ g mol^–1^) was extracted from tamarind seed powder (DSP Gokyo, Japan). *S. cerevisiae* cells were obtained from Dona Benta (Angel Yeast Co., China). Additional reagents included dimethyl sulfoxide (DMSO 99.5% v v^–1^; Sigma-Aldrich, USA), ethanol (99.5% v v^–1^; Da’Ilha, Brazil), sodium azide (NaN_3_; Sigma-Aldrich, USA), and sodium chloride (NaCl; Sigma-Aldrich, USA). Hydrochloric acid (HCl 37% v v^–1^) and sodium hydroxide (NaOH) (Synth, Brazil) were used for pH adjustment. Nile Blue (Sigma-Aldrich, USA) and fluorescein isothiocyanate (FITC) were used for confocal microscopy. All chemicals were of analytical grade. Ultrapure water (18.2 MΩ cm, Milli-Q, Millipore, USA) was used for all final sample preparations.

### 2.2 Polysaccharide Isolation

AMP was isolated by dispersion in a DMSO-water mixture (95:5, v v^–1^) at a concentration of 50 g L^–1^ under continuous magnetic stirring for 24 h. The dispersion was precipitated in ethanol (99.5% v v^–1^), filtered, and dried under vacuum at 30 °C. XG was obtained as reported by Hazt et al.^9^ tamarind seed powder was dispersed in distilled water (40 g L^–1^) containing 200 mg L^–1^ NaN_3_ for 24 h. Precipitation was performed using two volumes of ethanol (99.5% v v^–1^), followed by drying under the same conditions applied to AMP.

The mass-average molar mass (*M*_w_), number-average molar mass (*M*_n_), and dispersity (*D̵*) of AMP, XG, and FITC-labeled AMP (AMP–FITC) are presented in Table S1.

### 2.3 Preparation of *S. cerevisiae* Samples: Inactivated and Active Yeast

*S. cerevisiae* cells were prepared in two forms:

#### Thermally Inactivated Yeast (Y-IN)

Dry commercial yeast cells were suspended in distilled water (5 wt %) and thermally treated at 80 °C for 30 min under constant agitation (500 rpm). After cooling to 23 °C, the suspension was centrifuged five times (1000*g*, 5 min), discarding the supernatant each time. The final pellet was resuspended in ultrapure water to obtain a 17 wt % stock suspension.

#### Yeast Active (Y-AC)

Yeast cells were suspended in distilled water (5 wt %) under identical conditions but without thermal treatment.

### 2.4 Cell Viability

Yeast cell viability was assessed using methylene blue staining (0.001 g L^–1^), as described by O’Connor-Cox et al.^20^ After dilution in ultrapure water, 150 μL of the yeast suspension was mixed with equal volume of dye solution and incubated for 5 min at room temperature. Viable (unstained) and nonviable (blue-stained) cells were counted using a Neubauer chamber. Samples included active yeast in ultrapure water, viable yeast (2.00 wt %) suspended in polymer solutions of AMP (1.00 wt %) or XG (1.45 wt %), and Y-IN (2.00 wt %). For polymer-containing samples, cell counts were performed at 0, 24, 166 and 240 h.

### 2.5 Fourier-Transform Infrared-Spectroscopy

Fourier-transform infrared (FT-IR) spectra were acquired using an ALPHA FT-IR spectrometer (Bruker) equipped with an ATR module. Samples (Y-AC and Y-IN) were analyzed directly on ATR crystal over the 4000–400 cm^–1^ range, using 32 coadded scans at 4 cm^–1^ resolution. Spectra were baseline-corrected and normalized using Opus Viewer before assignment and comparative peaks analysis.

### 2.6 Zeta Potential

Zeta potential (ζ-potential) was measured by electrophoretic mobility at 25 °C using ZetaSizer Nano ZS (Malvern, UK) equipped with DTS1070 disposable cuvettes. Samples were diluted to 0.01 wt % in ultrapure water. Ionic strength effects were evaluated by titrating Y-IN suspensions with NaCl using an MPT-2 autotitrator. The pH dependence (pH 3.0 to 8.0) was determined for both Y-AC and Y-IN by adjusting the pH with 0.1 mol L^–1^ HCl or NaOH.

### 2.7 Potentiometric Titration

Titrations were performed using an HI901 automatic titrator (Hanna Instruments) equipped with a pH combination electrode. Approximately 800 mg of yeast cells (Y-AC and Y-IN) were dispersed in ultrapure water (0.05 wt %) and homogenized for 2 h at pH 3.0, acidified using a 0.10 mol L^–1^ aqueous HCl solution before analysis. Titrations were performed at 25 °C by dynamic incremental addition of standardized 0.104 mol L^–1^ aqueous NaOH solution, with automatic step adjustment based on signal stabilization. The titrant volume per step ranged from 0.03 to 0.50 mL, and the end point was set to pH 10.0. Blank titrations (ultrapure water/NaCl background) were subtracted. Apparent p*K*_a_ values and functional group distributions were determined by nonlinear fitting using R software.

The resulting acid–base profiles were used to assess proton-binding behavior and infer the distribution of ionizable functional groups on the yeast surface. Nonlinear fitting and extraction of apparent p*K*_a_ values were performed using the R language (RStudio 2024.12.1 + 563 software).

### 2.8 Phase diagram

Phase diagrams were constructed following the methodology described by Hazt et al.^9^ Mixtures were prepared using 50:50 wt % of stock solutions of XG (2 wt %) and AMP (10 wt %), prepared either in ultrapure water or in 20 mmol L^–1^ aqueous NaCl solution (9), both containing 200 mg L^–1^ NaN_3_. The mixtures were adjusted to the final volume with ultrapure water and vortexed for 1 min. Final concentrations ranged from 0.015 to 1 wt % for XG and from 0.075 to 5 wt % for AMP.

Phase separation was monitored over time at 25 ± 1 °C. The densities of the polysaccharide solutions were determined to be 0.994 g mL^–1^ for AMP and 0.976 g mL^–1^ for XG, with a margin of error of ±0.005 g mL^–1^. The phase diagram was constructed by plotting the equilibrium concentrations of each phase after complete separation, allowing the binodal curve and tie-lines to be defined along the 50:50 wt % axis. Additional mixtures were prepared along the binodal curve to validate the phase separation behavior (9) (Figure S1, Supporting Information).

### 2.9 Pickering Emulsions

AMP (12 wt %) and XG (3 wt %) were initially dispersed in ultrapure water containing 200 mg L^–1^ NaN_3_. The resulting stock dispersions were then diluted to prepare emulsions with different AMP/XG ratios. The main formulation (Point 3), consisting of 1.00 wt % AMP and 1.45 wt % XG, was used to incorporate Y-IN at concentrations ranging from 0.0 to 15.0 × 10^4^ cells mL^–1^. The mixtures were homogenized by vortexing for 60 s, and the final emulsions were prepared under three distinct ionic conditions: without NaCl, with 1.00 mmol L^–1^ NaCl, or with 20.0 mmol L^–1^ NaCl.

Phase separation was monitored over 10 days (24, 48, 144, and 240 h) at 22 ± 2 °C to assess colloidal stability. In addition, emulsions containing 6.0 × 10^4^ of Y-IN cells mL^–1^ were evaluated using alternative AMP/XG ratios: point 1 (1.00 wt % AMP and 1.30 wt % XG), point 2 (4.00 wt % AMP and 0.35 wt % XG), point 3 (1.00 wt % AMP and 1.45 wt % XG), and point 4 (4.00 wt % AMP and 0.52 wt % XG) (Figure S1, Supporting Information).

The formulation described in Point 3 was tested with 6.33 × 10^4^ Y-AC cells mL^–1^ to evaluate its behavior in comparison with the inactivated counterpart under identical conditions.

### 2.10 Confocal Laser Scanning Microscopy

AMP was fluorescently labeled with fluorescein isothiocyanate (FITC) following the protocol described by Belder and Granath.^21^ In brief, 1 g of AMP was dissolved in 10 mL of DMSO containing 200 μL of pyridine. To this, 0.1 g of FITC and 20 mg of dibutyltin dilaurate were added, and the reaction mixture was heated at 95 °C for 2 h. The resulting AMP-FITC conjugate was precipitated, washed with ethanol (99% v v^–1^) until all free FITC, not conjugated to AMP, was removed, and dried under vacuum at 80 °C.

Emulsions were formulated using AMP, with 10 wt % of the polysaccharide replaced by AMP-FITC. Y-AC and Y-IN particles were labeled with 5 mg L^–1^ Nile blue and stirred overnight to ensure uniform dye incorporation. Samples were mounted in glass-bottom cell culture dishes (Greiner Bio-One) and imaged using water immersion objectives (20× and 60×) on a Nikon A1R MP confocal laser scanning microscope. Fluorescence excitation was performed at 561 nm (FITC) and 663 nm (Nile blue). The contact angle between yeast cells and the AMP–XG interface was measured using ImageJ Fiji software. At 5 mg L^–1^, the fluorophore had no observable effect on phase separation, indicating that it did not influence the behavior of the system.

The lengths of 50 individual structures were measured under each experimental condition, and contact-angle measurements were obtained from replicates. Data are expressed as mean ± standard deviation. Differences among groups were evaluated by one-way analysis of variance (ANOVA), followed by Tukey’s multiple-comparison test, with statistical significance set at *p* < 0.05.

## 3 Results and Discussion

The aim of this study was to identify the physicochemical contribution of yeast particles to AMP–XG systems. To assess particle–polymer interactions both Y-AC and Y-IN cells were analyzed. This comparison allows to differentiate between purely physical stabilization (PE) and potential biological behaviors, identifying how thermal processing impacts the cell’s function as a stabilizer.

### 3.1 Characterization of *S. cerevisiae*

First, it was necessary to confirm the biological state of the samples. Thermal inactivation conditions (80 °C, 30 min) were chosen to ensure complete loss of cell viability, exceeding the *D*-value (decimal reduction time) for *S. cerevisiae* (3 min at 55 °C).^22^ As shown in Supporting Information (Table S2), the heat treatment was effective, resulting in zero viability for Y-IN. Importantly, Y-AC cells maintained consistent viability in biopolymer dispersions up to 240 h, confirming the biocompatibility in AMP–XG system.

Once the biological state was established, the physicochemical properties of the cell wall were characterized by FTIR, potentiometric titration, and ζ-potential. High temperatures are known to alter functional group accessibility and rearrange the yeast cell wall,^23^ potentially impacting surface charge and colloidal behavior.

Figure 1a shows the FTIR spectra of commercial and Y-IN. The peak at 3293 cm^–1^ corresponds to the overlapping of OH/NH group vibrations. The CH_2_ (methylene) groups appear at 2924 cm^–1^. The band at 1660 cm^–1^ (C=O) indicates the presence of amide I and carboxylate ion. Peaks at 1535 cm^–1^ and 1400 cm^–1^ correspond to NH (amide II) and carboxyl groups, respectively. Additionally, the P=O group is visible at 1040 cm^–1^. A previous study reported similar FTIR bands for Y-IN *S. cerevisiae*.^24^ Although the spectral profile remained similar, subtle but systematic differences in peak intensity were observed, particularly in the amide and phosphate regions, indicating changes in protein and phosphorylated groups after thermal exposure. These differences are consistent with reports that heating promotes partial denaturation of mannoproteins and alteration of the outer layer.^25,26^

**Figure 1:**
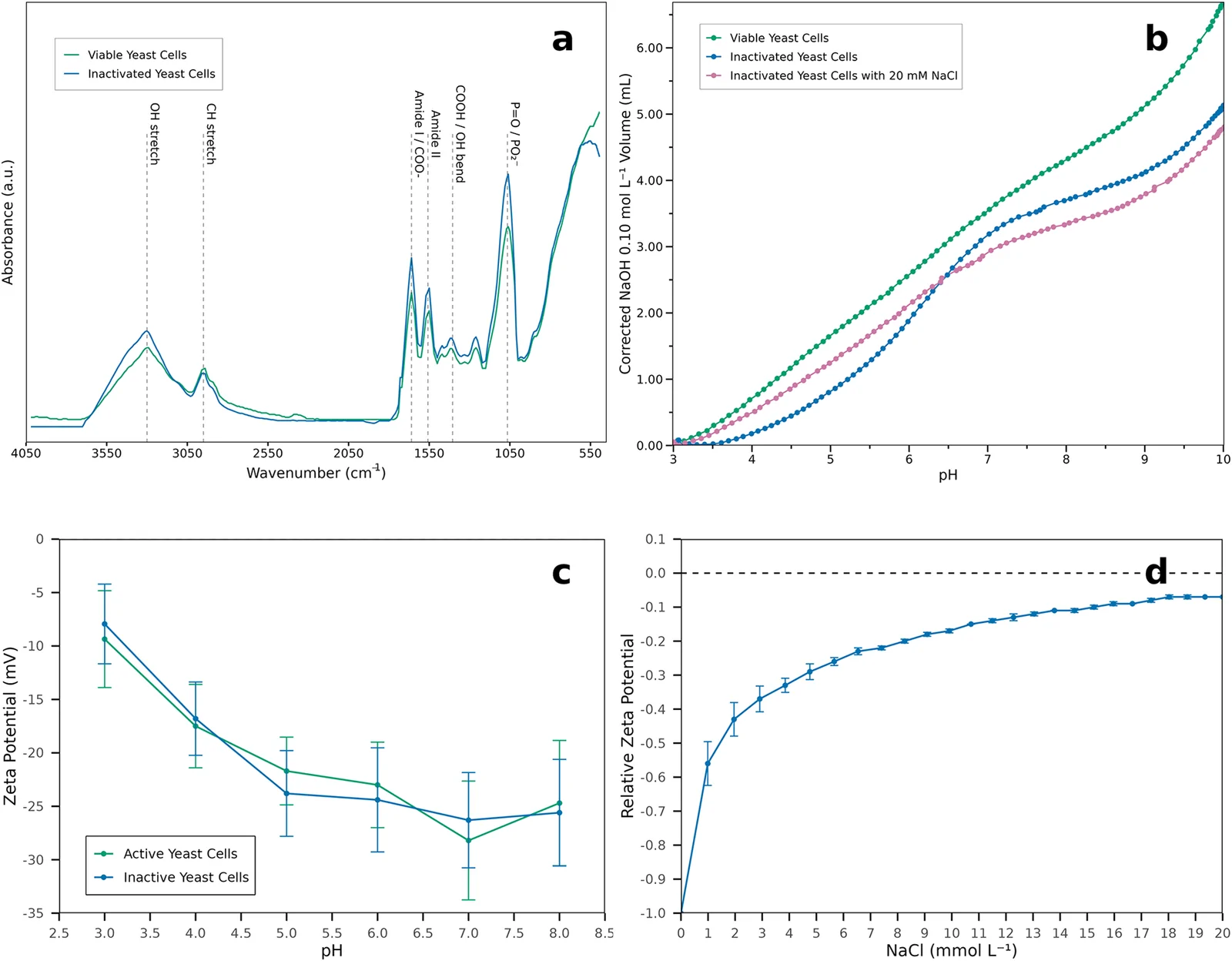
Physicochemical characterization of viable (Y-AC) and thermally inactivated (Y-IN) *Saccharomyces cerevisiae* cells: (a) FTIR spectra showing changes in functional groups after thermal inactivation. (b) Potentiometric titration curves indicating shifts in titratable groups due to heat treatment and aqueous NaCl solution. (c) ζ-Potential vs pH for Y-AC and Y-IN, showing consistently negative surface charges regardless of inactivation. (d) ζ-Potential of Y-IN at increasing NaCl concentrations, highlighting electrostatic screening and reduced surface charge at higher ionic strength.

To quantify these changes, potentiometric titration was performed, as shown in Figure 1b, revealing a multisite system of weak acids.^27,28^ Table 1 describes the estimated p*K*_a_ values for carboxyl, phosphate, and amide/hydroxyl groups, which are typical for fungal cell walls.^29,30^

**Table 1:** Apparent p*K*_a_ Values and Relative Fractions of Titratable Functional Groups (Carboxyl, Phosphate, and Amide/Hydroxyl) Determined From Potentiometric Titration of Viable (Y-AC), Thermally Inactivated (Y-IN), and Y-IN in 20 mmol L^–1^ Aqueous NaCl Solution^a^

	Y-AC	Y-IN	Y-IN with 20 mmol L^–1^ NaCl
p*K*_a_ 1	4.51 ± 0.10	4.77 ± 0.09	4.51 ± 0.10
carboxyl sites fraction (%)	32 ± 3	32 ± 3	36 ± 3
p*K*_a_ 2	6.60 ± 0.06	6.36 ± 0.05	6.35 ± 0.07
phosphate sites fraction (%)	23 ± 3	50 ± 4	27 ± 5
p*K*_a_ 3	9.11 ± 0.13	9.27 ± 0.12	9.21 ± 0.14
amide/hydroxyl sites fraction (%)	33 ± 5	36 ± 5	31 ± 5

a Values are reported as fitted ±standard error obtained from nonlinear least-squares fitting of the potentiometric titration curves.

Thermal inactivation increased the apparent contribution of phosphate groups to the total titratable sites from 23% in Y-AC to 50% in Y-IN. This change suggests that thermal treatment altered the organization of the cell–wall components, increasing the apparent accessibility or ionization of phosphate-containing groups. Because no direct structural measurements were performed, this result should not be interpreted as definitive evidence of cell–wall dehydration or mannoprotein folding. When 20 mmol L^–1^ NaCl was added, the apparent phosphate contribution decreased to 27%, since Na^+^ ions can associate with negatively charged phosphate groups, partially neutralize surface charge, and reduce the apparent density of ionized phosphate sites.

FTIR and potentiometric titration demonstrated structural changes, but ζ-potential measurements at different pH values did not record any significant variation in Y-AC and Y-IN values (Figure 1c). The samples had a negative charge during each pH evaluation due to the model containing high amounts of carboxyl and phosphate groups from the cellular wall. This suggests that thermal inactivation does not result in changes to the intrinsic nature of the cellular wall but rather, to the organization of the cellular wall, i.e., protein denaturation and group conformation, as thermal inactivation does not disrupt the acid–base balance and therefore the electrokinetic mobility is stable at all pH evaluations even after thermal inactivation.

However, the ionic strength of the medium has a significant impact on the surface charge. Figure 1d illustrates the behavior of Y-IN in the presence of NaCl. The ions can neutralize the charge, forming an electrostatic screening. A NaCl concentration of 1 mmol L^–1^ is sufficient to reduce the ζ-potential by approximately 50%, while 20 mmol L^–1^ largely suppresses the repulsive forces between particles. From the perspective of utilizing yeast cells to stabilize emulsions, surface charge plays a critical role. In Pickering emulsions, particles with a high ζ-potential usually repel one another, and when positioned at the emulsion interface, they promote droplet repulsion, preventing coalescence. Conversely, uncharged particles tend to agglomerate, increasing emulsion instability.^31,32^

The combined characterization results from the cell wall suggest that thermal inactivation does not modify the underpinning acid–base chemistry, explaining the comparable ζ-potential behavior at different pH levels. Nonetheless, heat exposure alters the physical organization and arrangement of the groups for optimizing the accessibility of the phosphate groups, which makes the system remarkably sensitive to ionic strength. These parameters directly influence the particle–interface interactions and are therefore expected to have a decisive impact on the performance of Y-IN as stabilizers in AMP–XG Pickering emulsions.

### 3.2 Stability of Pickering Emulsions stabilized by Y-IN Particles

In Figure S2 (Supporting Information), four AMP/XG formulations are presented (point 1: AMP 1.0/XG 1.3; point 2: AMP 1.0/XG 0.35; point 3: AMP 1.0/XG 1.45; point 4: AMP 4.0/XG 0.52), all stabilized with 6.0 × 10^4^ Y-IN cells mL^–1^ under different salinity conditions (no NaCl, 1 mmol L^–1^ NaCl, and 20 mmol L^–1^ NaCl). The pH of all emulsions remained within the range of 5.5–6.0, while the temperature was maintained at 22 ± 2 °C throughout the experimental period.

In the systems without salt and with 1 mmol L^–1^ NaCl, the sediment initially consists mainly of Y-IN cells. However, over time, partial emulsion destabilization occurs, leading to the accumulation of destabilized droplets at the bottom of the system. Under 20 mmol L^–1^ NaCl, the reduction of the cell surface charge significantly compromises emulsion stability within the first 24 h, resulting in the formation of sediment composed of aggregated cells and coalesced emulsion droplets.

Points 2 and 4 exhibit a distinct behavior. In these systems, the upper fraction appears to be emulsified and is predominantly associated with XG dispersed in AMP-rich phase, whereas the lower fraction contains precipitated material that is more closely related to AMP and yeast cells (Figure S3 Supporting Information).

Point 3 was selected for the main graphs because its higher XG content results in increased viscosity, and promotes the formation of a more robust polymeric network, with higher interfacial tension. This combination enhances the resistance of the emulsion to phase separation, allowing a clearer evaluation of the effects of salinity and yeast concentration. Therefore, point 3 represents the most stable and suitable formulation for quantitative analysis. The other formulations, although presented in Figure S2, exhibit instabilities or phase-inversion phenomena, making direct comparisons less reliable.

The effect of Y-IN on AMP–XG emulsions was investigated under three salinity conditions: without aqueous NaCl solution, 1 mmol L^–1^ aqueous NaCl solution, and 20 mmol L^–1^ aqueous NaCl solution. In the absence of salt, turbidity increased proportionally with Y-IN concentration, especially within the first 24 h. Sedimentation began during this period and became more evident over time. In systems without Y-IN, phase separation was only observed after 144 h. Emulsions with lower Y-IN cells (1.5 and 4.5 × 10^4^ cells mL^–1^) showed less sedimentation, while higher concentrations (up to 6.0 × 10^4^ cells mL^–1^) resulted in a greater emulsified fraction, indicating that increasing yeast content enhances emulsion stability. Figure 2 illustrates the sedimentation behavior of AMP–XG emulsions containing different concentrations of Y-IN under varying aqueous NaCl solution conditions over time.

**Figure 2:**
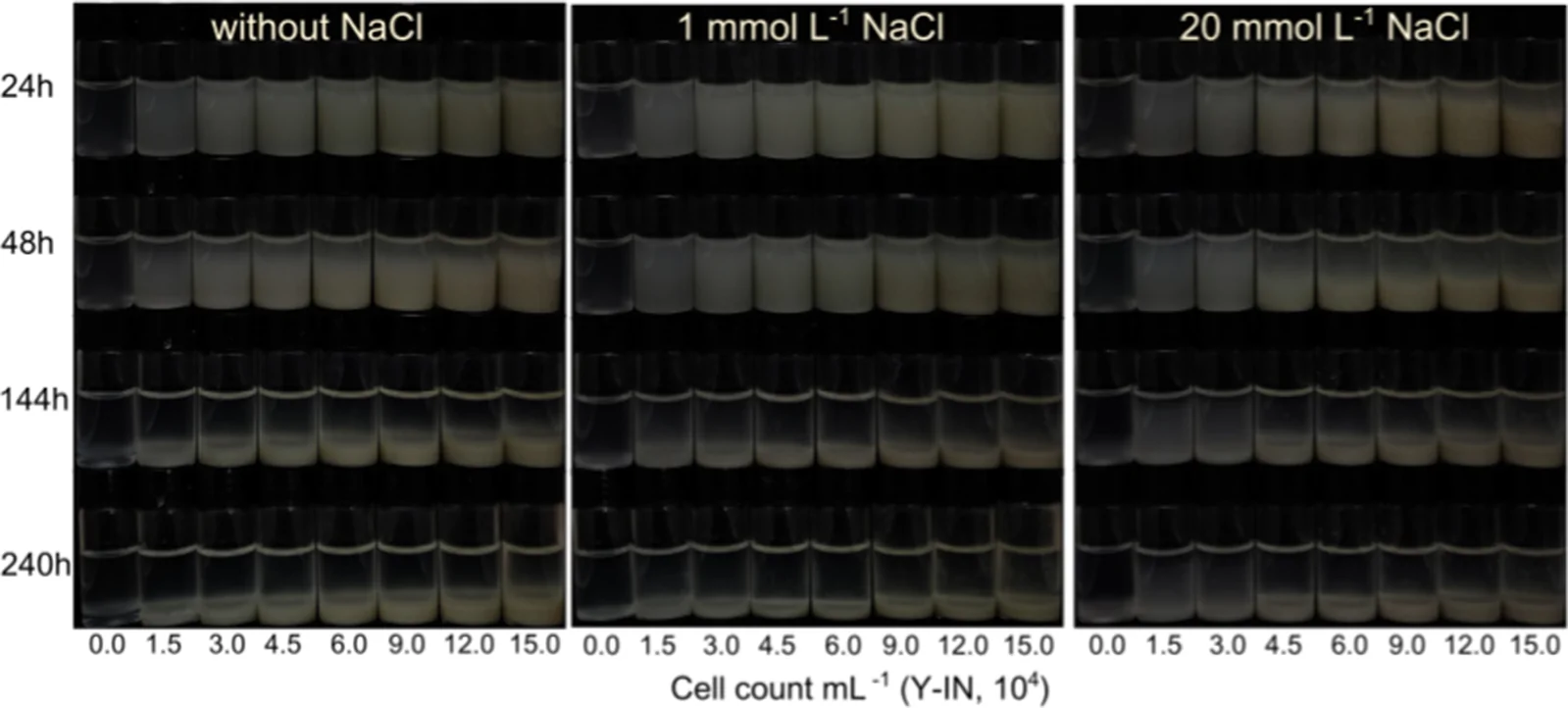
Macroscopic evaluation of AMP (1.00 wt %) in XG (1.45 wt %) with varying concentrations of Y-IN particles (0.0 to 15.0 × 10^4^ cells mL^–1^) over time (24, 48, 144, and 240 h), under three conditions: without aqueous NaCl solution, 1.0 mmol L^–1^ aqueous NaCl solution, and 20 mmol L^–1^ aqueous NaCl solution.

The behavior of yeast cells under these conditions is illustrated in Figure 2. The addition of 1 mmol L^–1^ aqueous NaCl solution, which reduces approximately 50% of the yeast charges according to ζ-potential measurements, did not significantly alter phase separation compared to salt-free samples, although slightly lower emulsified fractions were observed. However, at 20 mmol L^–1^ aqueous NaCl solution, a pronounced effect was noted: after 24 h, emulsions containing 6.0 to 15.0 × 10^4^ Y-IN cells mL^–1^ began to sediment. This process intensified at higher concentrations, such as 15.0 × 10^4^ cells mL^–1^, and in 48 h, even samples with lower Y-IN content showed signs of sedimentation.

In summary, the presence of aqueous NaCl solution significantly affects the stability of AMP–XG emulsions containing Y-IN, with higher salt concentrations accelerating phase separation and compromising particle dispersion, especially in systems with elevated yeast content.

These results are corroborated by the confocal microscopy images in Figure 3, which depict Y-IN samples with cell counts of 3.0, 6.0, and 12.0 × 10^4^ cells mL^–1^. As the Y-IN concentration increases, a greater density and distribution of fluorescent particles is observed, with red fluorescence being predominant. The 3.0 × 10^4^ cells mL^–1^ Y-IN sample shows a less uniform dispersion, with particles distributed sparsely along the droplet interfaces, reflecting a less cohesive structure. In contrast, the 6.0 × 10^4^ cells mL^–1^ Y-IN sample exhibits a homogeneous film of particles coating the droplet interfaces, indicating greater interfacial organization. The 12.0 × 10^4^ cells mL^–1^ Y-IN sample displays a dense network of particles distributed both at the interface and within the continuous phase, which directly correlates with the higher turbidity and sedimentation observed in macroscopic analyses.

**Figure 3:**
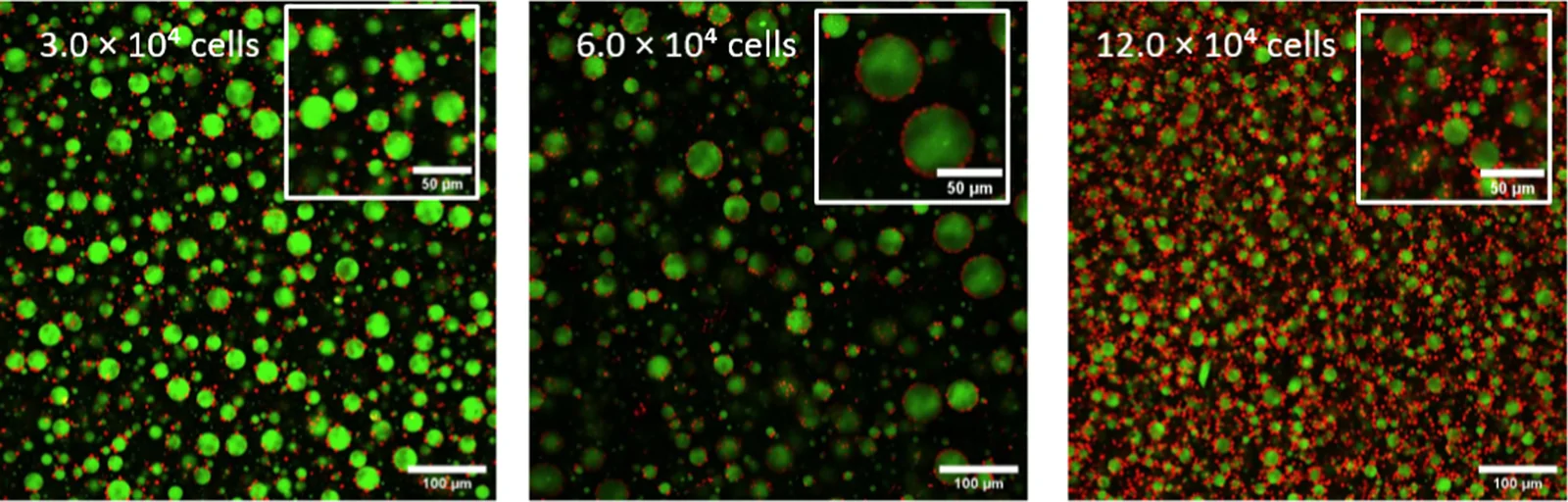
Confocal laser scanning microscopy images of AMP (1.00%) in XG (1.45%) emulsions without added aqueous NaCl solution, containing 3.0, 6.0, and 12.0 × 10^4^ Y-IN cells mL^–1^ obtained after 24 h of emulsion preparation. Scale bars: 100 μm (main images), 50 μm (insets).

Figure 4 shows the vertical distribution of yeast cells in AMP–XG Pickering emulsions. The cells, marked in red, are predominantly positioned at the interface of the green AMP droplets, forming stable structures. A gradient is observed along the height of the emulsion: in the upper region (“top”), the droplets are smaller and exhibit more discrete cell coverage, reflecting lower density and mass. In the middle region (“middle”), there is a higher concentration of droplets with more uniform yeast coverage, indicating greater interfacial organization. At the base of the emulsion (“bottom”), the droplets are larger and densely coated with cells, which is directly associated with the sedimentation observed at the macroscopic scale.

**Figure 4:**
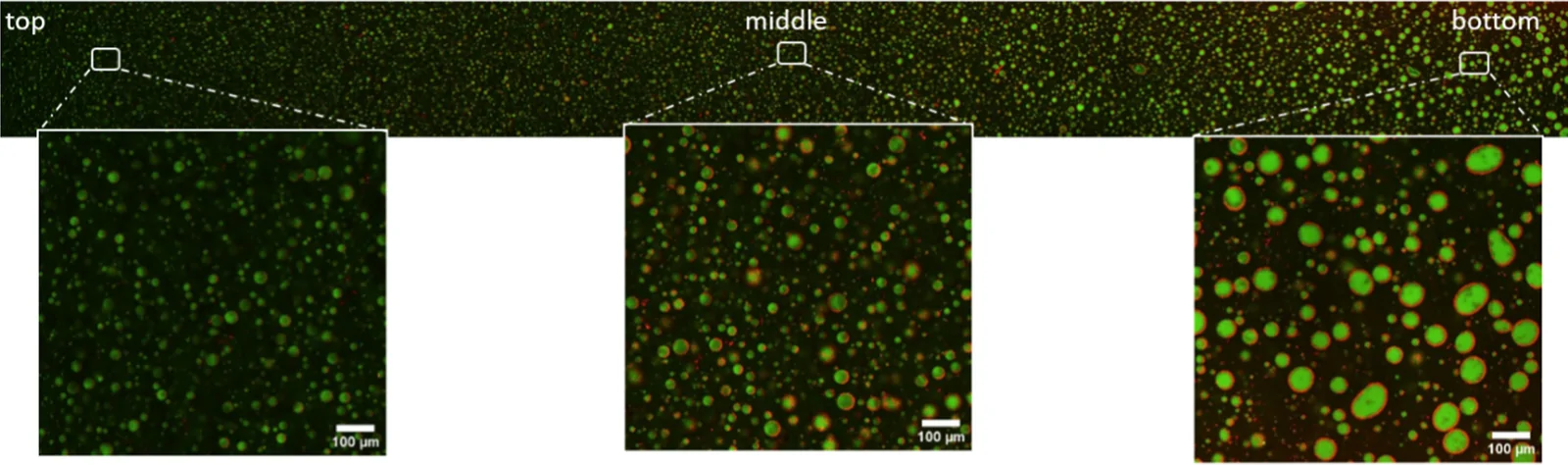
Confocal laser scanning microscopy images of AMP (1.00%) in XG (1.45%) emulsions without added NaCl, containing 6.0 × 10^4^ Y-IN cells mL^–1^ and obtained after 24 h of preparation. The images illustrate the vertical distribution of yeast cells within the emulsion, highlighting structural differences across the top, middle, and bottom regions. Scale bars: 100 μm (main images), 50 μm (insets).

This vertical profile (Figure 4) confirms that increasing cell concentration enhances interfacial coverage and reinforcing protection against coalescence. The ζ-potential contributes to system stability by preventing cell aggregation and maintaining particle dispersion. However, even with this electrostatic stability, sedimentation occurs because of the volume and mass of the formed structures, as evidenced in the image. This behavior highlights the importance of spatial particle distribution in the overall stability of emulsions.

To investigate the influence of salt on emulsion behavior, a concentration of 6.0 × 10^4^ Y-IN cells mL^–1^ was selected because it produced the most stable system. As shown in Figure 5, in the absence of NaCl, Y-IN cells (4.6 ± 0.6 μm) were predominantly located at the droplet interface, maintaining the Pickering structure. At 1.0 mmol L^–1^ NaCl, the cells (4.5 ± 0.7 μm) remained at the interface, preserving the Pickering configuration. In contrast, at 20 mmol L^–1^ NaCl, the cells (4.7 ± 0.5 μm) were predominantly located within the dispersed AMP phase, resulting in the loss of interfacial stabilization. Cell size did not differ significantly among the water, 1.0 mmol L^–1^ NaCl, and 20 mmol L^–1^ NaCl conditions (*p* > 0.05; *n* = 50). Therefore, the salt-induced destabilization cannot be attributed to changes in cell size but rather to the redistribution of Y-IN cells from the interface to the AMP phase.

**Figure 5:**
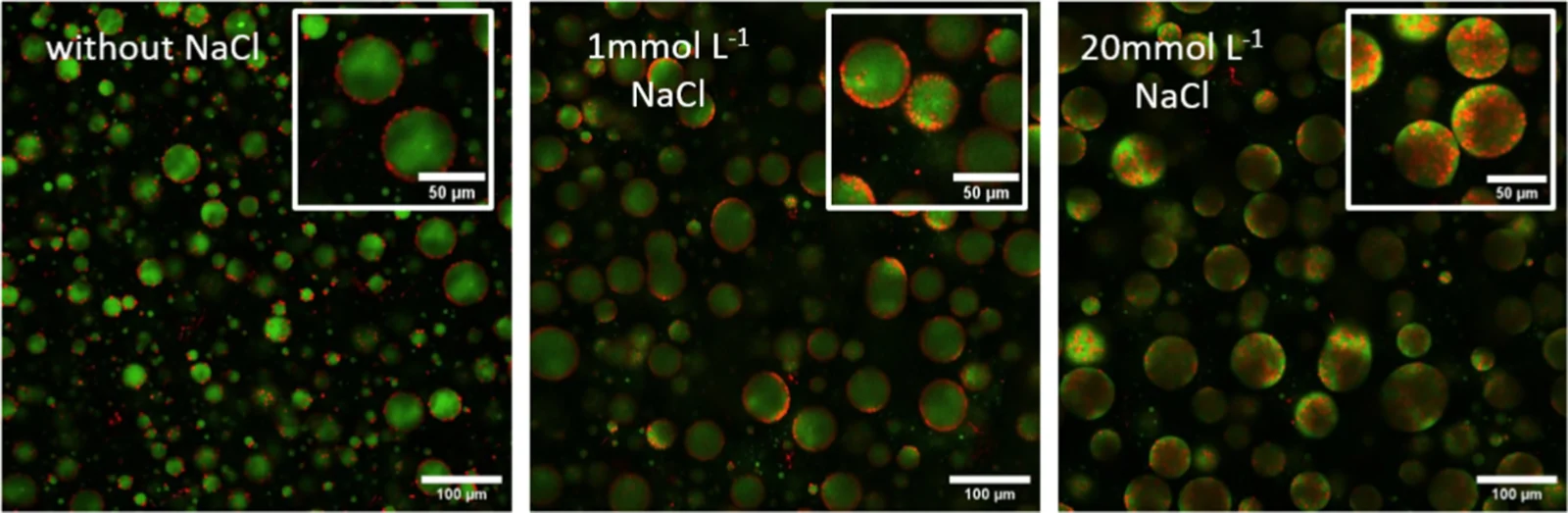
Confocal laser scanning microscopy images of AMP (1.00%) in XG (1.45%) emulsions containing 6.0 × 10^4^ Y-IN cells mL^–1^ under different NaCl concentrations were obtained after 24 h of preparation: without aqueous NaCl solution, 1.0 mmol L^–1^ aqueous NaCl solution, and 20.0 mmol L^–1^ aqueous NaCl solution. Scale bars: 100 μm (main images), 50 μm (insets).

This shift reflects a change in phase affinity due to surface charge screening, as AMP and XG lack titratable groups.^8^ The 3D confocal microscopy reconstruction (Figure S4, Supporting Information) visualizes this internalization process, confirming that the loss of emulsion stability is directly caused by the detachment of cells from the interface (breakdown of the Pickering effect).

### 3.3 Stability of Pickering Emulsions by Y-AC Particles

The stabilization of AMP emulsions by active yeast cells (Y-AC) was evaluated using confocal microscopy under different aqueous NaCl solution concentrations. In the absence of salt, Y-AC cells, marked in red, are continuously distributed around the AMP droplets, forming well-defined Pickering emulsions. This uniform interfacial coverage contributes to the structural stability of the droplets, preventing coalescence and maintaining dispersion over time. As illustrated in Figure 6, the addition of 1 mmol L^–1^ aqueous NaCl solution promotes greater cell coverage around the droplets. At higher salt concentrations (20 mmol L^–1^), the droplets become smaller and more irregular, with yeast cells migrating into the interior of the droplets.

**Figure 6:**
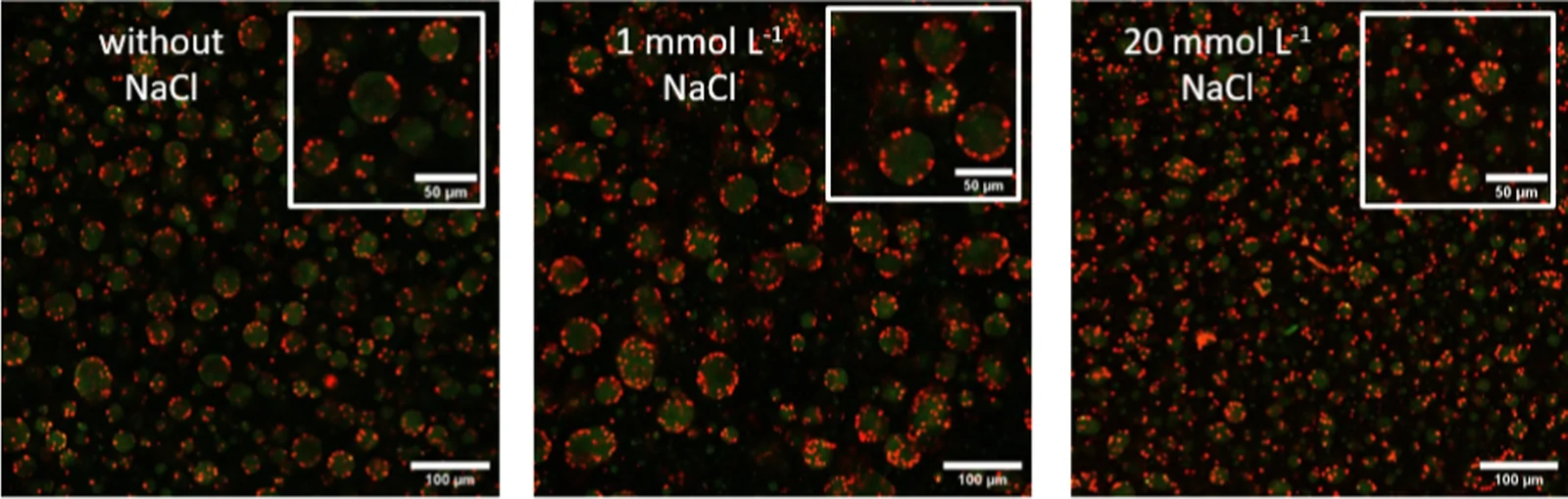
Confocal laser scanning microscopy images of AMP (1.00%) in XG (1.45%) emulsions containing 6.33 × 10^4^ Y-AC cells mL^–1^ under different aqueous NaCl solution concentrations were obtained after 24 h of preparation: water (without NaCl), 1.0 mmol L^–1^ aqueous NaCl solution, and 20.0 mmol L^–1^ aqueous NaCl solution. Scale bars: 100 μm (main images), 50 μm (insets).

The results from CLSM are coherent with the particle wettability results when evaluating the contact angle (Figure S5, Supporting Information). The contact angle represents the relative affinity for one of the phases: the larger the angle, the larger the affinity for the dispersed phase (AMP). In water (without NaCl), Y-IN exhibit an angle of 89° ± 11°, as demonstrated via the particles at the interface of the system. The value increases slightly to 116° ± 10°, ° in the presence of 1 mmol L^–1^ of aqueous NaCl solution. In this example, the yeast continues to attach to the interface, but the affinity toward the AMP dispersed phase has increased. With the addition of 20 mmol L^–1^ of aqueous NaCl solution, no interfaces are observed, and all the yeast migrates to the AMP phase. A similar trend is observed for active yeast (Y-AC), by increasing the salt concentration in the same proportions; the angle is increased from 114 ± 18° to 130° ± 7°. At the highest salt concentration tested (20 mmol L^–1^), fewer particles are attached to the interface. The contact angle increased significantly in the presence of NaCl for both active and inactivated yeast (*p* < 0.05). For active yeast, no significant difference was observed between 1 and 20 mmol L^–1^ NaCl (*p* > 0.05).

Compared with other stabilizers used in AMP–XG emulsions, β-lactoglobulin microgels require approximately 1.0 wt % and are effective mainly at pH 4–5,^9^ whereas cellulose nanoparticles are used at 0.15–0.50 wt % and stabilize the system through either XG-mediated viscosity enhancement or partial interfacial adsorption.^10^ Here, *S. cerevisiae* cells formed an interfacial layer at 6.0 × 10^4^ cells mL^–1^ under salt-free and low-salt conditions. Although larger than these nanoparticles, yeast cells require no particle synthesis or surface modification and may add biological functionality to the emulsion.

## 4 Conclusion

This study demonstrates, for the first time, that *S. cerevisiae*, in both Y-AC and Y-IN states, can effectively stabilize amylopectin-xyloglucan (AMP–XG) water-in-water (W/W) Pickering emulsions. Comprehensive physicochemical analyses revealed that thermal inactivation does not alter the fundamental acid–base chemistry of the yeast cell wall but significantly reorganizes its structure, leading to increased accessibility of phosphate groups and enhanced sensitivity to ionic strength. ζ-Potential and titration data confirmed that electrostatic screening induced by NaCl strongly modulates interfacial behavior, with high ionic strength suppressing particle repulsion and driving yeast migration toward the AMP-rich phase.

Stability assays showed that Y-IN cell counts ≥6.0 × 10^4^ cells mL^–1^ promote robust interfacial adsorption and delay phase separation, forming Pickering layers around AMP droplets. Confocal microscopy corroborated these findings, revealing that both Y-IN and Y-AC can anchor at the polymer–polymer interface and form well-organized interfacial films in low-salt environments. However, the displacement of yeast into the dispersed phase at 20 mmol L^–1^ aqueous NaCl solution confirmed that ionic strength is a critical factor controlling particle wettability, phase affinity, and long-term stability. Contact-angle measurements further supported a salt-induced shift in wettability, reflecting decreased interfacial anchoring and compromised stabilization.

## Supplementary Material



## Notes

HPSEC characterization and macromolecular parameters (*M*_w_, *M*_n_, and dispersity) of amylopectin (AMP), FITC-labeled amylopectin (AMP-FITC), and xyloglucan (XG) (Table S1); AMP/XG phase diagrams in water and in the presence of NaCl (Figure S1); viability of active (Y-AC) and inactivated (Y-IN) *S. cerevisiae* in water and biopolymer dispersions (Table S2); macroscopic stability of AMP/XG water-in-water emulsions containing Y-IN cells at different NaCl concentrations over time (Figure S2); confocal laser scanning microscopy images of AMP/XG emulsions stabilized by Y-IN cells in the absence and presence of NaCl (Figure S3); 3D confocal microscopy reconstruction showing yeast-cell distribution at the AMP/XG interface and in the bulk phase (Figure S4); contact-angle measurements of active and inactivated yeast cells at the AMP/XG interface in the absence and presence of NaCl (Figure S5) (PDF)

## References

[1] Esquena J. (2016). Water-in-water
(W/W) emulsions. Curr. Opin. Colloid Interface
Sci..

[2] Nicolai T., Murray B. (2017). Particle stabilized
water in water emulsions. Food Hydrocoll..

[3] Niang S., Lee H. J., Benyahia L., Nicolai T., Renou F., Ferji K. (2025). Synthesis
and design of double-hydrophilic copolymers
for dextran/PEO water-in-water emulsion stabilization. Carbohydr. Polym..

[4] Aveyard R., Binks B. P., Clint J. H. (2003). Emulsions
stabilised solely by colloidal
particles. Adv. Colloid Interface Sci..

[5] Akbari S., Nour A. H. (2021). Emulsion types,
stability mechanisms and rheology:
A review. Int. J. Innov. Res. Sci. Stud..

[6] Hazt B., Pereira P. G., Do Amaral L. F. M., Gallina P. R., Martin S., Hess Gonçalves O. (2023). Unconventional and conventional
Pickering emulsions: Perspectives and challenges in skin applications. Int. J. Pharm..

[7] Kong Y., Chen J., Hong Z., Guo R., Huang Q. (2025). Insights into
the Pickering emulsions stabilized by yeast dietary fiber: Interfacial
adsorption kinetics, rheological characteristics, and stabilization
mechanisms. Food Chem..

[8] De
Freitas R. A., Nicolai T., Chassenieux C., Benyahia L. (2016). Stabilization of Water-in-Water Emulsions by Polysaccharide-Coated
Protein Particles. Langmuir.

[9] Hazt B., Bassani H. P., Elias-Machado J. P., Aldinucci Buzzo J. L., Silveira J. L. M., De Freitas R. A. (2020). Effect
of pH and protein particle
shape on the stability of amylopectin–xyloglucan water-in-water
emulsions. Food Hydrocoll..

[10] Pires C., Régnier B. M., Dos Santos M. J. R., Alves De Freitas R. (2023). Effect of
sulfate-ester content and nanocellulose allomorph on stability of
amylopectin-xyloglucan water-in-water emulsions. Food Hydrocoll..

[11] Li Y., Zhang J., Ao S., Cao H., Li Y., Li B. (2025). Synergistic co-encapsulation
of Saccharomyces cerevisiae
and Lactobacillus helveticus in water-in-water Pickering emulsion
with enhanced probiotic viability and digestive resistance. Food Biosci..

[12] Ao S., Sun Y., Ding X., Zhou C., Wang Y., He J. (2025). Water-in-water
Pickering emulsions as a fascinating culture and embedding
medium for Saccharomyces cerevisiae. Food Biosci..

[13] Nejadmansouri M., Eskandari M. H., Yousefi G. H., Riazi M., Hosseini S. M. H. (2023). Promising
application of probiotic microorganisms as Pickering emulsions stabilizers. Sci. Rep..

[14] Firoozmand H., Rousseau D. (2016). Microbial cells as
colloidal particles: Pickering oil-in-water
emulsions stabilized by bacteria and yeast. Food Res. Int..

[15] Srivastava S. P., Singh H. D., Baruah J. N., Krishna P. V., Iyengar M. S. (1970). Physicochemical
studies on the water-yeast cells-gas-oil system. J. Appl. Chem..

[16] Zhu W., Ma W., Li C., Pan J., Dai X., Gan M. (2014). Magnetic molecularly imprinted microspheres via yeast stabilized
Pickering emulsion polymerization for selective recognition of λ-cyhalothrin. Colloids Surf. A Physicochem. Eng. Asp..

[17] Fu D., Fu J., Xu H., Shao Z., Zhou D., Zhu B. (2024). Glycation-induced
enhancement of yeast cell protein for improved
stability and curcumin delivery in Pickering high internal phase emulsions. Int. J. Biol. Macromol..

[18] Firoozmand H., Rousseau D. (2014). Tailoring the morphology
and rheology of phase-separated
biopolymer gels using microbial cells as structure modifiers. Food Hydrocoll..

[19] Xie H., Zhao W., Ali D. C., Zhang X., Wang Z. (2021). Interfacial
biocatalysis in bacteria-stabilized Pickering emulsions for microbial
transformation of hydrophobic chemicals. Catal.
Sci. Technol..

[20] O’Connor-Cox, E. ; Lodolo, E. J. ; Axcell, B. C. Methylene Blue Staining: Use at Your Own Risk. Technical Quarterly-Master Brewers Association of the Americas, 1997; Vol. 34, pp 306–312.

[21] Belder A. N., Granath K. (1973). Preparation and properties of fluorescein-labelled
dextrans. Carbohydr. Res..

[22] López-Malo A., Guerrero S., Alzamora S. M. (1999). Saccharomyces
cerevisiae Thermal
Inactivation Kinetics Combined with Ultrasound. J. Food Prot..

[23] Klis F. M., Mol P., Hellingwerf K., Brul S. (2002). Dynamics of cell wall structure in
Saccharomyces cerevisiae. FEMS Microbiol. Rev..

[24] Hlihor R. M., Diaconu M., Fertu D., Chelaru C., Sandu I., Tavares T. (2013). Bioremediation of Cr­(VI)
Polluted Wastewaters by Sorption
on Heat Inactivated Saccharomyces cerevisiae Biomass. Int. J. Environ. Res..

[25] Salari R., Bazzaz B. S. F., Rajabi O., Khashyarmanesh Z. (2013). New aspects
of Saccharomyces cerevisiae as a novel carrier for berberine. Daru J. Pharm. Sci..

[26] Ene I. V., Walker L. A., Schiavone M., Lee K. K., Martin-Yken H., Dague E., Gow N. A. R., Munro C. A., Brown A. J. P. (2015). Cell
Wall Remodeling Enzymes Modulate Fungal Cell Wall Elasticity and Osmotic
Stress Resistance. mBio.

[27] Narong P., James A. E. (2006). Effect of pH on the ζ-potential
and turbidity
of yeast suspensions. Colloids Surf. A Physicochem.
Eng. Asp..

[28] Stratford M., Steels H., Nebe-von-Caron G., Novodvorska M., Hayer K., Archer D. B. (2013). Extreme resistance
to weak-acid preservatives
in the spoilage yeast Zygosaccharomyces bailii. Int. J. Food Microbiol..

[29] Naeem A., Woertz J. R., Fein J. B. (2006). Experimental
Measurement of Proton,
Cd, Pb, Sr, and Zn Adsorption Onto the Fungal Species Saccharomyces
Cerevisiae. Environ. Sci. Technol..

[30] Rogowska A., Pomastowski P., Złoch M., Railean-Plugaru V., Król A., Rafińska K., Szultka-Młyńska M., Buszewski B. (2018). The influence of different pH on the electrophoretic
behaviour of Saccharomyces cerevisiae modified by calcium ions. Sci. Rep..

[31] Boostani S., Hosseini S. M. H., Golmakani M. T., Marefati A., Abdul Hadi N. B., Rayner M. (2020). The influence of emulsion
parameters on physical stability
and rheological properties of Pickering emulsions stabilized by hordein
nanoparticles. Food Hydrocoll..

[32] McClements D. J., Jafari S. M. (2018). Improving emulsion formation, stability
and performance
using mixed emulsifiers: A review. Adv. Colloid
Interface Sci..

