# Supplementary material for: Stabilization of Amylopectin–Xyloglucan Water-in-Water Pickering Emulsions by Yeast Cells
Source: ACS Omega. 2026 Aug 29;11(36):54313–21. doi: 10.1021/acsomega.6c05131 (PMC13584770; doi:10.1021/acsomega.6c05131)
Supplement: Supplementary file 1 [file ao6c05131_si_001.pdf]

Stabilization of Amylopectin–Xyloglucan Water-in-Water Pickering Emulsions by Yeast Cells

Cassiano Pires[1#], Lilian Fernanda Martins do Amaral[1#], Natalia Brasil Posselt Costa[1], Joslaine Jacumazo[1], Jaqueline Carneiro[1], Lazhar Benyahia[2] & Rilton Alves de Freitas[1*]

[1] BIOPOL, Center for Development and Innovation in Materials and Biomaterials-CDIM, Curitiba, 80210-170, Brazil

[2] IMMM, UMR 6283 CNRS - Le Mans Université, Avenue Olivier Messiaen, Le Mans, 72085, France

[#]Contributed equally to this paper.

[*]Corresponding author. E-mail address: rilton@ufpr.br

Size-Exclusion Chromatography

The mass-average molar mass (M_w), number-average molar mass (M_n), and dispersity (Đ) of AMP, AMP-FITC and XG were determined by high-performance size-exclusion chromatography (HPSEC). Samples were prepared at 1 mg mL^{-1} in an aqueous solution of 0.1 mol L^{-1} $NaNO_3$ containing 200 mg L^{-1} sodium azide and subsequently filtered through 0.22 µm cellulose ester membranes (Millipore) prior to injection. Analyses were performed using an HPSEC system equipped with a multi-angle static laser light scattering detector (LS, model DSP-F, Wyatt Technology, Santa Barbara, CA, USA), a refractive index detector (RI, Waters 2410, Milford, MA, USA), and a UV detector (Pharmacia LKB, Uvicord VW 2251, λ = 280 nm). The chromatographic system (Waters) was coupled to four Ultrahydrogel® gel-permeation columns (2000, 500, 250, and 120) connected in series, with exclusion limits of 7×10^6, 4×10^5, 8×10^4, and 5×10^3 g mol^{-1}, respectively. The elution temperature was maintained at 21 °C. The mobile phase consisted of an aqueous solution of 0.1 mol L^{-1} $NaNO_3$ containing 200 mg L^{-1} sodium azide. Data acquisition and processing were performed using the ASTRA software (version 4.70.07, Wyatt Technology). The refractive index increment (dn/dc) values used were 0.146 mL g^{-1} for AMP and 0.108 mL g^{-1} for XG as reported by Hazt et al. (2020) (1).

Table S1. Macromolecular parameters: mass-average molar mass (M_w), number-average molar mass (M_n), and dispersity (Đ) of the biopolymers amylopectin (AMP), amylopectin with fluorescein isothiocyanate (AMP-FITC), and xyloglucan (XG), as determined by HPSEC.

**Parâmetros**	**AMP**	**AMP-FITC**	**XG**
**M_w / g mol^{-1}**	3.0×10^7	1.1×10^7	1.2×10^6
**M_n / g mol^{-1}**	2.8×10^7	7.8×10^6	1.0×10^6
**Đ (Mw/Mn)**	1.07	1.39	1.17

Phase diagram of mixtures between amylopectin and xyloglucan with water and NaCl.

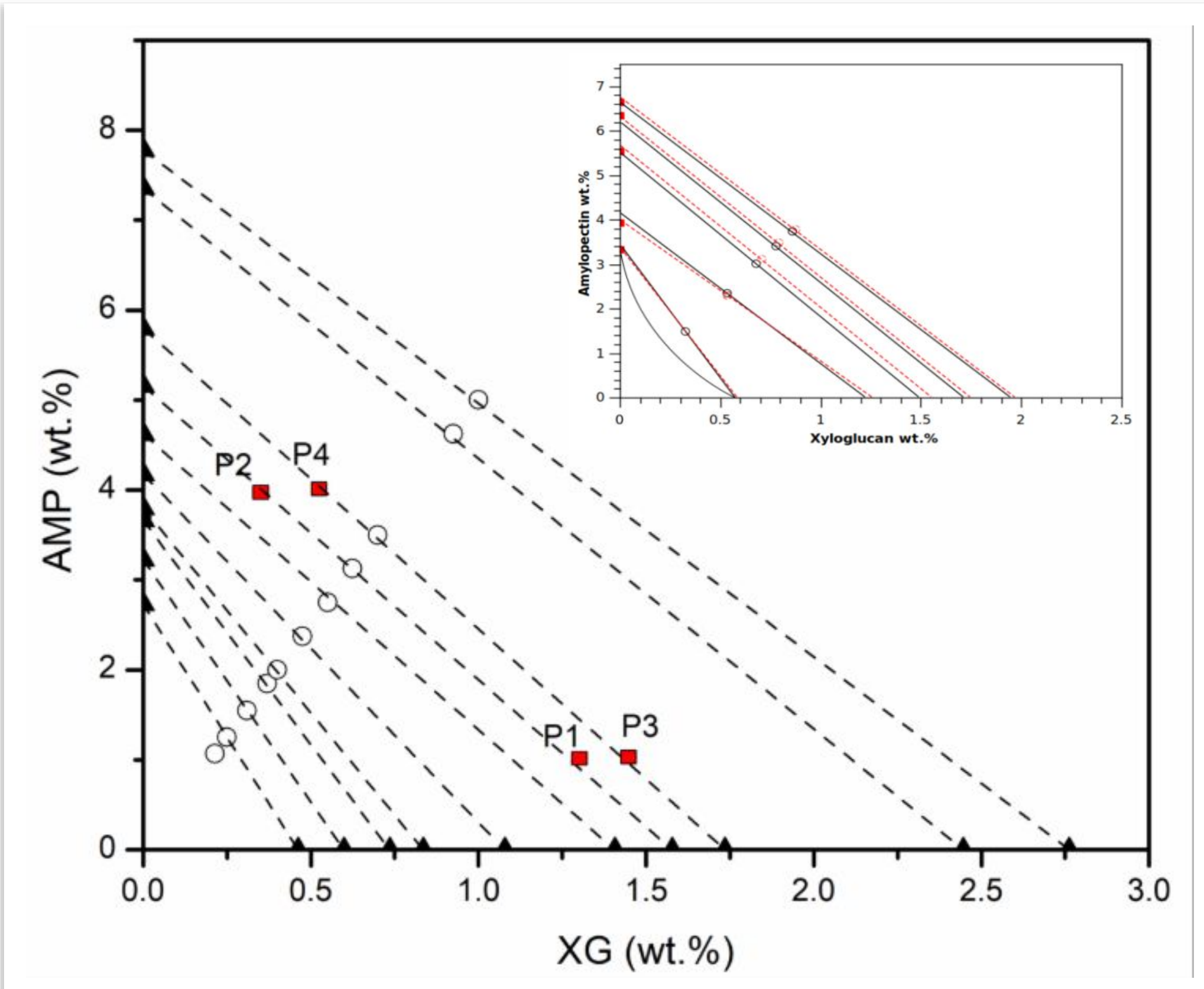

Figure S1. Phase diagram of mixtures between AMP and XG. (P1) Point 1 (AMP 1.00 wt.%, XG 1.30 wt.%), (P2) Point 2 (AMP 1.00 wt.%, XG 0.35 wt.%), (P3) Point 3 (AMP 1.00 wt.%, XG 1.45 wt.%), and (P4) Point 4 (AMP 4.00 wt.%, XG 0.52 wt.%). Inserted: Phase diagram of mixtures between AMP and XG. Without sodium chloride (**black line**) and in the presence of 20 mmol L^{-1} of salt (**red line**) at 25 ^{o}C.

Viability of active (Y-AC) and inactivated (Y-IN) *Saccharomyces cerevisiae*

Table S2. Viability assessment of 2 wt.% Saccharomyces cerevisiae in water and biopolymer dispersions at 0 and 24 h.

Medium	Time (h)	Viable Cell (10^4) mL $^{-1}$	Non-Viable Cells (10^4) mL $^{-1}$	Total Count (10^4) mL $^{-1}$	Viability (%)
Water (control)	0	4.91	1.42	6.33	**77.6**
Amylopectin	0	1.68	1.37	3.05	**55.1**
	24	1.79	1.20	2.99	**59.9**
	144	1.57	1.26	2.83	**55.5**
	240	1.60	1.42	3.02	**52.9**
Xyloglucan	0	2.60	1.24	3.84	**67.7**
	24	2.65	1.38	4.03	**65.8**
	144	2.70	1.21	3.91	**69.0**
	240	2.79	1.01	3.80	**73.4**
Inactivated Yeast	24	0.00	5.95	5.95	**0**

Yeast viability ranged from 65.8% to 73.4% in xyloglucan and from 52.9% to 59.9% in amylopectin over 240 h. Relative to the initial values, the maximum changes were 5.7 and 4.8 percentage points, respectively, which were within the estimated counting error of approximately 5.8% associated with evaluating 300 cellular elements using a Neubauer chamber.

Conventional *S. cerevisiae* lacks sufficient extracellular enzymatic activity to efficiently degrade these polysaccharides (Knox et al., 2004; Wang et al., 2024). Thus, the maintenance of cell viability should not be interpreted as evidence that the polymers were utilized as carbon sources. Instead, the cells may have persisted in a low-metabolic or quiescent state, consistent with the ability of *S. cerevisiae* to survive prolonged nutrient limitation (Gresham et al., 2011).

Macroscopic assays of amylopectin and xyloglucan mixtures in the absence of salt, with 1 and 20 mmol L^{-1} aqueous NaCl solution containing 6.0×10^4 Y-IN cells mL^{-1}.

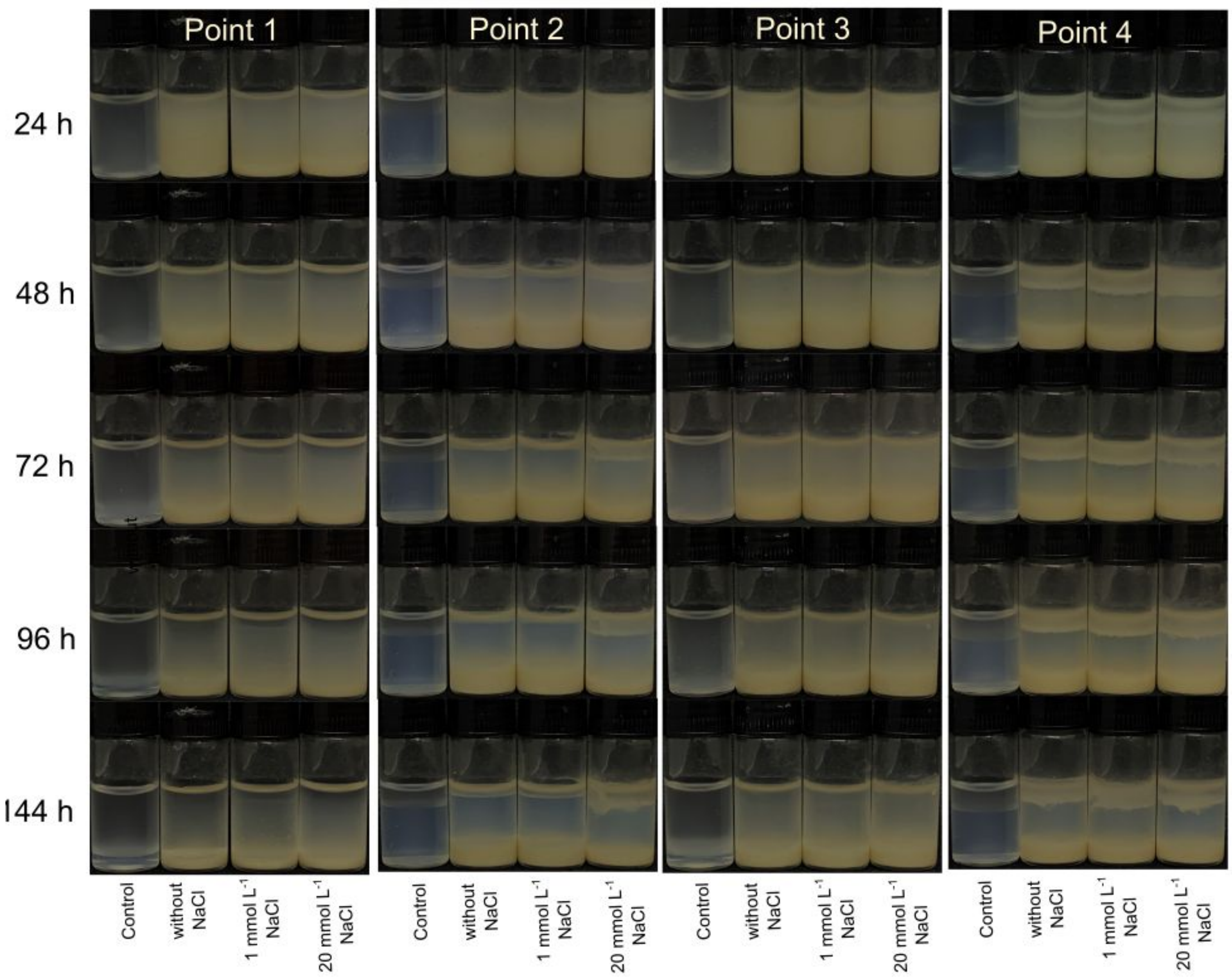

Figure S2. Macroscopic evaluation of AMP/XG formulations stabilized with 6.0×10^4 Y-IN cells particles over time (24 h, 48 h, 72 h, 96 h, and 144 h), across four experimental setups: Point 1 (AMP 1.00 wt.%, XG 1.30 wt.%), Point 2 (AMP 1.00 wt.%, XG 0.35 wt.%), Point 3 (AMP 1.00 wt.%, XG 1.45 wt.%), and Point 4 (AMP 4.00 wt.%, XG 0.52 wt.%). Samples were tested: Control, without NaCl, 1 mmol L^{-1} aqueous NaCl solution and 20 mmol L^{-1} aqueous NaCl solution.

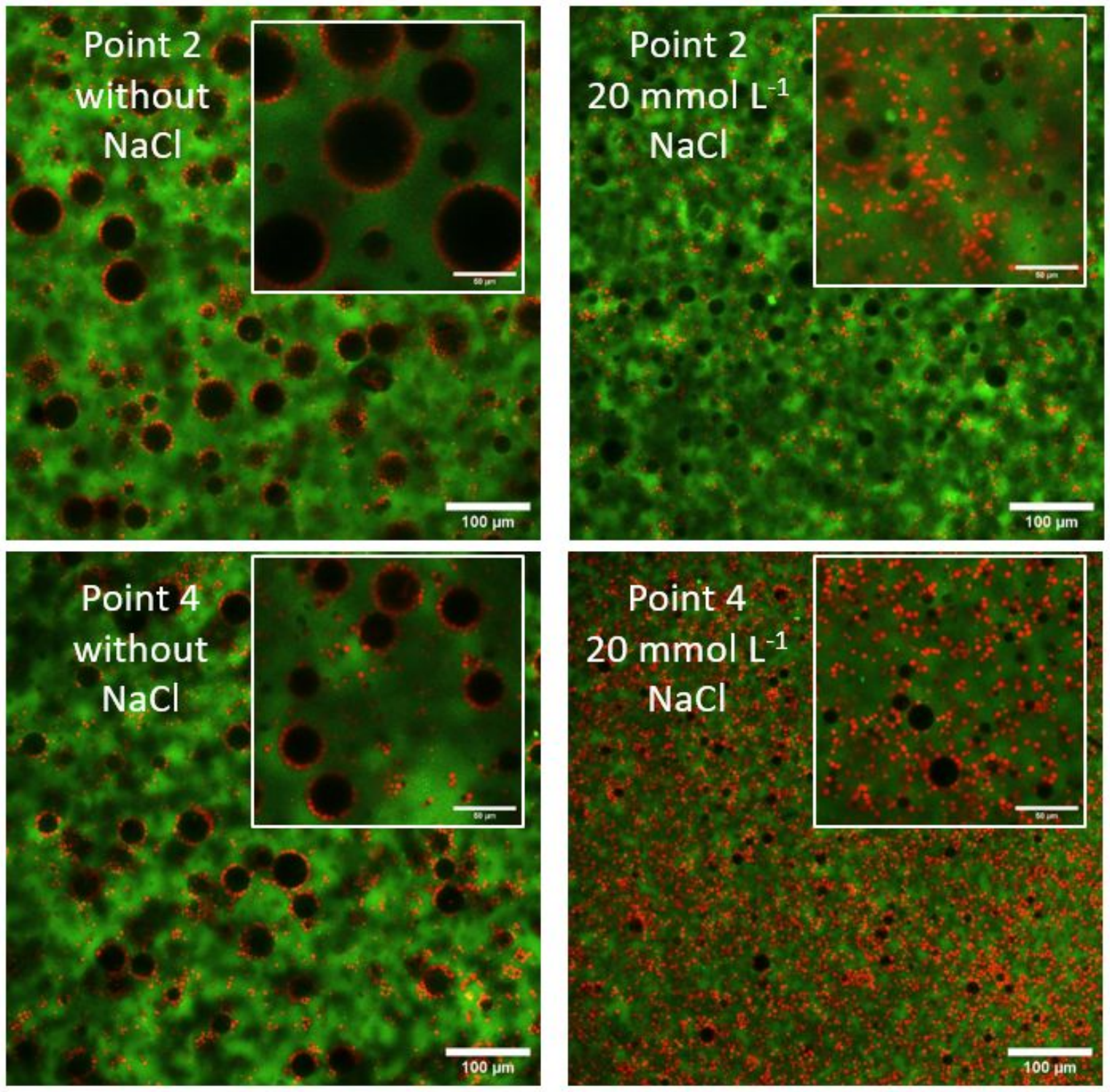

Figure S3. Confocal microscopy of Point 2 (4.00 wt.% AMP and 0.35 wt.% XG) and Point 4 (4.00 wt.% AMP and 0.52 wt.% XG), stabilized with 6.0×10^4 Y-IN cells particles over time (24 h)

3D Confocal microscopy

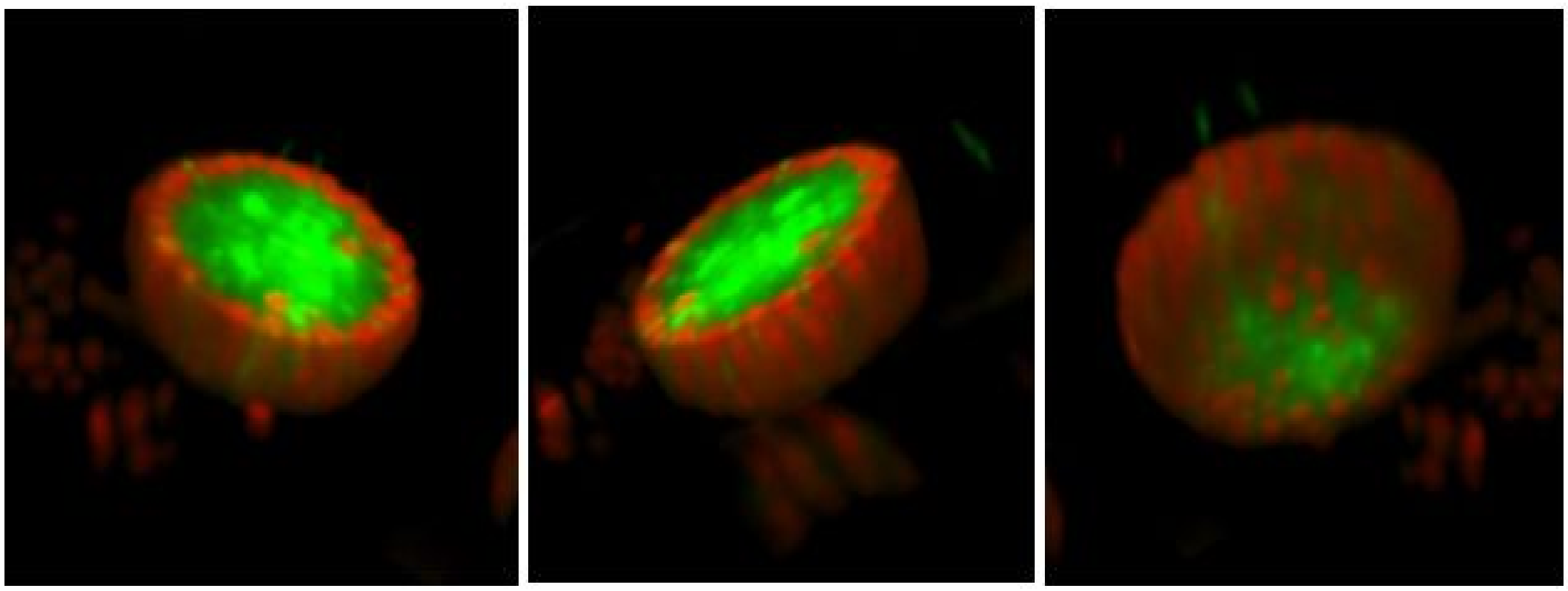

Figure S4. 3D confocal microscopy reconstruction. The visualization reveals yeast cells migrating from the interface into the bulk AMP phase at elevated ionic strength. This internalization confirms the detachment of the solid particles, marking the breakdown of the Pickering stabilization mechanism.

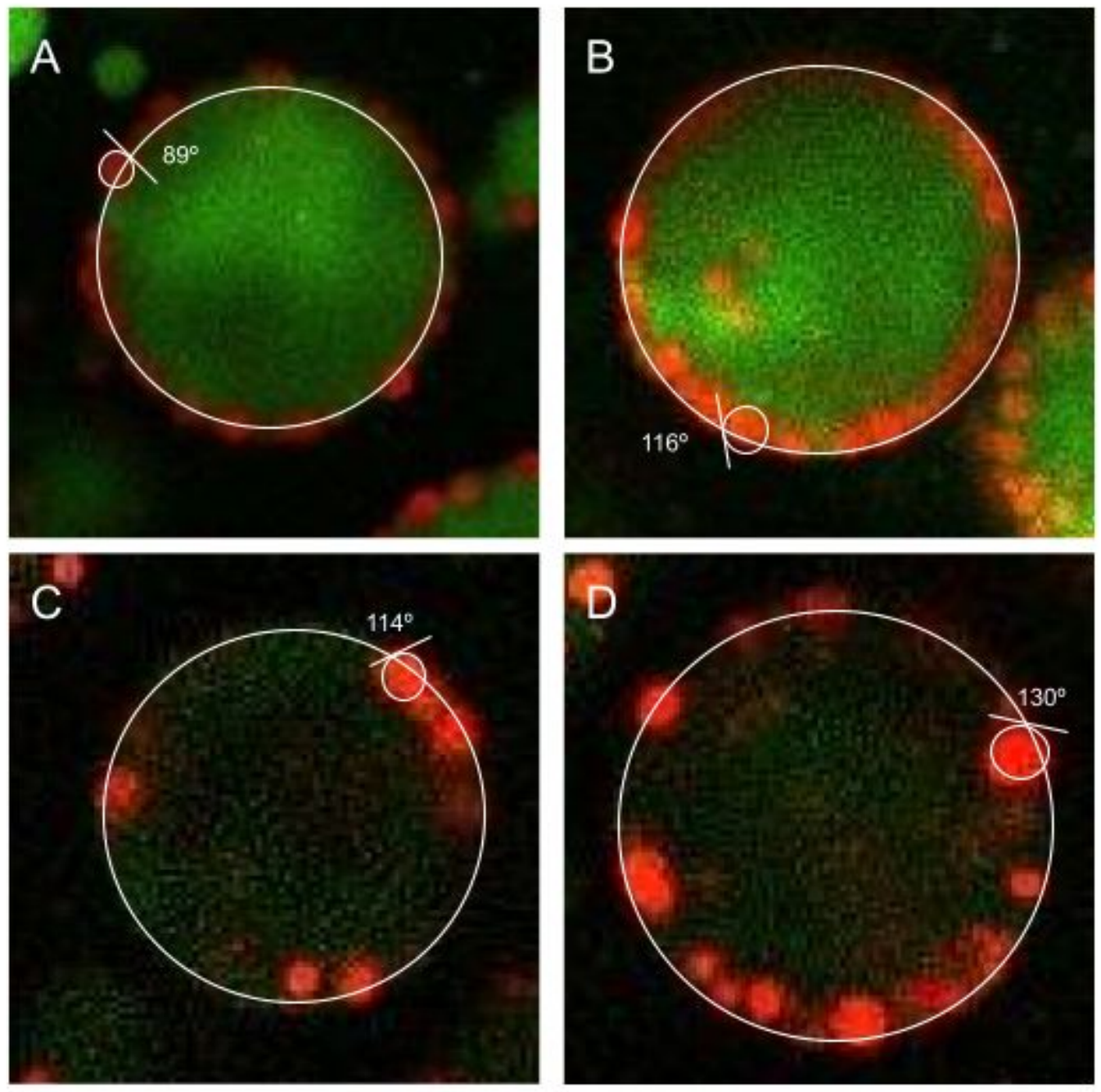

Figure S5. Contact-angle measurements obtained from confocal laser scanning microscopy images of AMP (1.00% w/w)-in-XG (1.45% w/w) water-in-water emulsions containing 6.0×10^4 yeast cells mL^{-1}, 24 h after preparation: (A) Y-IN in water; (B) Y-IN in 1.0 mmol L^{-1} NaCl; (C) Y-AC in water (without NaCl), and (D) Y-AC in 1.0 mmol L^{-1} NaCl.

Reference

1 - Hazt B, Bassani HP, Elias-Machado JP, Aldinucci Buzzo JL, Silveira JLM, De Freitas RA. Effect of pH and protein particle shape on the stability of amylopectin–xyloglucan water-in-water emulsions. Food Hydrocoll. 2020 July; 104:105769.

2- Knox AM, du Preez JC, Kilian SG. Starch fermentation characteristics of *Saccharomyces cerevisiae* strains transformed with amylase genes from *Lipomyces kononenkoae* and *Saccharomycopsis fibuligera*. Enzyme Microb Technol. 2004;34(5):453–460. https://doi.org/10.1016/j.enzmictec.2003.12.010

3- Gresham D, Boer VM, Caudy A, Ziv N, Brandt NJ, Storey JD, et al. System-level analysis of genes and functions affecting survival during nutrient starvation in *Saccharomyces cerevisiae*. Genetics. 2011;187(1):299–317. https://doi.org/10.1534/genetics.110.120766

4- Wang X, Guo N, Hu J, Gou C, Xie X, Zheng H, et al. Construction of an amylolytic *Saccharomyces cerevisiae* strain with high copies of α-amylase and glucoamylase genes integration for bioethanol production from sweet potato residue. Front Microbiol. 2024;15:1419293. https://doi.org/10.3389/fmicb.2024.1419293
